# A Comprehensive Approach to Identification of Surface-Exposed, Outer Membrane-Spanning Proteins of *Leptospira interrogans*

**DOI:** 10.1371/journal.pone.0006071

**Published:** 2009-06-29

**Authors:** Marija Pinne, David A. Haake

**Affiliations:** 1 Research Service, 151, Veterans Affairs Greater Los Angeles Healthcare System, Los Angeles, California, United States of America; 2 Division of Infectious Diseases, 111F, Veterans Affairs Greater Los Angeles Healthcare System, Los Angeles, California, United States of America; 3 Department of Medicine, The David Geffen School of Medicine at University of California at Los Angeles, Los Angeles, California, United States of America; Charité-Universitätsmedizin Berlin, Germany

## Abstract

Leptospirosis is a zoonosis with worldwide distribution caused by pathogenic spirochetes belonging to the genus *Leptospira*. The leptospiral life cycle involves transmission via fresh water and colonization of the renal tubules of their reservoir hosts or infection of accidental hosts, including humans. Bacterial outer membrane proteins (OMPs), particularly those with surface-exposed regions, play crucial roles in virulence mechanisms of pathogens and the adaptation to various environmental conditions, including those of the mammalian host. Little is known about the surface-exposed OMPs in *Leptospira*, particularly those with outer membrane-spanning domains. Herein, we describe a comprehensive strategy for identification and characterization of leptospiral transmembrane OMPs. The genomic sequence of *L. interrogans* serovar Copenhageni strain Fiocruz L1–130 allowed us to employ the β-barrel prediction programs, PRED-TMBB and TMBETA-NET, to identify potential transmembrane OMPs. Several complementary methods were used to characterize four novel OMPs, designated OmpL36, OmpL37, OmpL47 and OmpL54. In addition to surface immunofluorescence and surface biotinylation, we describe surface proteolysis of intact leptospires as an improved method for determining the surface exposure of leptospiral proteins. Membrane integration was confirmed using techniques for removal of peripheral membrane proteins. We also demonstrate deficiencies in the Triton X-114 fractionation method for assessing the outer membrane localization of transmembrane OMPs. Our results establish a broadly applicable strategy for the elucidation of novel surface-exposed outer membrane-spanning proteins of *Leptospira*, an essential step in the discovery of potential virulence factors, diagnostic antigens and vaccine candidates.

## Introduction

The etiologic agents of leptospirosis, pathogenic *Leptospira* spp., have a significant impact on public health throughout the developing world [1]–[3]. Many animals, especially rodents, serve as reservoir hosts in the transmission of pathogenic *Leptospira spp*. to humans. Exposure of mucous membranes or broken skin to water or soil contaminated with leptospires shed in animal urine can lead to a potentially fatal infection, characterized by jaundice, renal failure, and/or pulmonary hemorrhage [1], [3], [4]. Large outbreaks of leptospirosis occur in tropical and subtropical regions after heavy rainfall and dispersal of leptospires in contaminated water [2], [5]. One approach to infection control involves vaccines based on lipopolysaccharide (LPS), which dominates the leptospiral cell surface and can elicit protective immunity [6], [7]. However, leptospiral LPS is highly variable; its variations are thought to be the major antigenic determinant defining differences between approximately 230 serovars and contributing to serovar specific immunity [7]. In contrast, leptospiral outer membrane proteins (OMPs) are generally well conserved [8], [9] and would have the potential advantage of inducing comprehensive immunity [10].

Transmembrane OMPs are essential in maintaining the bacterial cell structure, attachment to various substrates, importing nutrients, and exporting bactericidal and toxic agents [11]. Thus, identification of OMPs is essential for the understanding of bacterial structure, function, interactions with the environment, and in the development of diagnostic and protective antigens for leptospirosis. The two major types of OMPs, outer membrane lipoproteins and transmembrane OMPs, differ significantly in their structure and how they are associated with the outer membrane. Lipoproteins become associated with membranes via a hydrophobic interaction between the N-terminal lipid moiety (three fatty acids) and the lipid bilayer phospholipids [8], [9]. In contrast, transmembrane OMPs are typically integrated into the lipid bilayer by amphipathic β-sheets arranged in a barrel-like structure [12], [13]. The genomes of several *Leptospira* strains have been sequenced [14], [15], [16], facilitating the application of bioinformatic algorithms to identify candidate OMPs, including lipoproteins [17] and transmembrane OMPs [18], [19]. Lipoproteins can be localized to one or more of four cellular compartments: the periplasmic leaflet of the inner membrane, the periplasmic or outer leaflets of the outer membrane, or external to the outer membrane [8], [9]. Notably, the bioinformatic algorithm, SpLip, is suitable for prediction of lipidation of spirochetal proteins but does not address the cellular destination of lipoproteins [17]. The low density of transmembrane spanning proteins (typically β-barrel proteins) in spirochetal outer membranes is striking [20], [21], with experimental evidence for only one such protein, OmpL1, having been thoroughly described in *Leptospira* spp. [22]–[24]. Several transmembrane OMPs have been described in other spirochetes, including borrelial Oms28 [25], P13 [26]–[28], BBA01 [29], P66 [30], [31], Oms38 [32], and BesC [33], and treponemal Msp [34], [35]. Moreover, genome sequence analysis suggests that many OM-spanning proteins of *Leptospira* spp. await discovery [15].

Our goal was to develop a comprehensive strategy for identification and characterization of novel outer membrane-spanning proteins in *Leptospira*. Leptospiral OMP identification has relied on subcellular fractionation methods, including Triton X-114 detergent extraction-phase partitioning and the isolation of OM vesicles [36]–[39]. These approaches have worked well for the differentiation of OM from inner membrane lipoproteins [36], [40]. However, the effectiveness of these approaches for the identification of transmembrane OMPs has not been thoroughly investigated. In fact, it has been shown that a majority of the OmpL1 porin is retained within the protoplasmic cylinder phase after Triton X-114 fractionation [22]. The structural characteristics of leptospiral transmembrane OMPs are predicted to be distinct from lipoproteins, potentially limiting the application of existing cellular fractionation methods. Therefore, a strategy involving several complementary methods has been employed to identify and characterize novel transmembrane OMPs of *L. interrogans*.

Four candidate nonlipoprotein OMPs selected *in silico* were found to be integral, surface-exposed OMPs using surface immunofluorescence, surface biotinylation, surface proteolysis, and membrane affinity methods. These data support their designation as OmpL36, OmpL37, OmpL47 and OmpL54. Unlike outer membrane lipoproteins, these transmembrane OMPs were poorly solubilized by Triton X-114, indicating that detergent-based methods may not be suitable for the fractionation of leptospiral transmembrane OMPs. Herein, we present an alternative strategy for defining and identifying integral OMPs by employing multiple methods to experimentally verify outer membrane integration and surface exposure. It is anticipated that this comprehensive approach will facilitate the identification of novel transmembrane OMPs with the potential to serve as virulence factors, new serodiagnostic antigens and vaccine candidates.

## Materials and Methods

### Bacterial strains and growth conditions

*L. interrogans* serovar Copenhageni strain Fiocruz L1–130 [5] was cultivated at 30°C in Probumin™ Vaccine Grade Solution (84-066-5, Celliance™ Kankakee, IL) diluted five-fold into autoclaved distilled water. Competent *E. coli* NEB 5-α (New England Biolabs, Ipswich, MA), and BLR(DE3)pLysS (Novagen, Madison, WI) were used for cloning and expression, respectively. *E. coli* were grown in Luria-Bertani (LB) broth or on agar plates with 50 µg/ml carbenicillin, 12.5 µg/ml tetracycline or 34 µg/ml chloramphenicol (Sigma-Aldrich, St. Louis, MO) when appropriate.

### *In silico* identification of *L. interrogans* outer membrane proteins

The following algorithms were used to identify candidate transmembrane OMPs in *L. interrogans* serovar Copenhageni strain Fiocruz L1–130 [15], [41]. Online versions of the SignalP 3.0 (http://www.cbs.dtu.dk/services/SignalP) [42] and LipoP 1.0 (http://www.cbs.dtu.dk/services/LipoP/) [43] programs were used to discriminate between lipoprotein and other protein signal peptides. Alpha-helical transmembrane domains were detected using the TMHMM version 2.0 (http://www.cbs.dtu.dk/services/TMHMM). The SpLip algorithm [17] was utilized to identify and eliminate lipoproteins. Transmembrane OMPs were identified using two β-barrel prediction programs, PRED-TMBB (http://biophysics.biol.uoa.gr/PRED-TMBB/) [18] and TMBETA-NET (http://psfs.cbrc.jp/tmbeta-net/) [19]. Four genes were chosen for further studies based on the following criteria: (i) presence of a signal peptide lacking a lipoprotein signal peptidase (SPII) cleavage site, (ii) absence of inner membrane-spanning α-helices other than the signal peptide, and (iii) prediction of at least six membrane-spanning β-strands by either PRED-TMBB or TMBETA-NET [18], [19].

### Cloning, expression and purification of recombinant OmpL36, OmpL37, OmpL47 and OmpL54

The genomic loci and the proposed names for the genes in parentheses are: Lic13166 *(ompL36)*, Lic12263 *(ompL37)*, Lic13050 *(ompL47)*, and Lic13491 *(ompL54)*. The genes encoding the four predicted OMPs were cloned into the expression vector, pET-20b(+) (Novagen). All primer sequences for amplification from Fiocruz L1–130 DNA are listed in Table 1. PCR was performed with Phusion High Fidelity DNA Polymerase (Finnzymes, Woburn, MA) and the following conditions for amplification of *ompL36*, *ompL37* and *ompL47*: 98°C for 30 sec, 30 cycles at 98°C for 10 sec, 59°C for 30 sec, 72°C for 30 sec, followed by 72°C for 7 min and cooling to 4°C. PCR conditions to amplify *ompL54* were: 98°C for 30 sec, 30 cycles at 98°C for 10 sec, 67°C for 30 sec, 72°C for 1 min 20 sec, followed by 72°C for 7 min and cooling to 4°C. PCR products were digested with *Nde*I and *Xho*I or *Nde*I and *Hind*III (New England Biolabs) for *ompL37*, *ompL47* and *ompL54* or *ompL36*, respectively and ligated to pET-20b(+) digested with either *Nde*I and *Xho*I or *Nde*I and *Hind*III. The plasmids were used to transform *E. coli* NEB 5-α and purified using the QIAprep Spin Miniprep Kit (Qiagen, Valencia, CA). After confirming the presence of correct inserts by restriction enzyme digestion, the plasmids were used to transform competent *E. coli* BLR(DE3)pLysS. Cultures were grown to OD_600_ ∼0.5 and then protein expression was induced with 0.4 mM isopropyl-β-D-thiogalactopyranoside. The His-tagged OmpL36, OmpL37 and OmpL47 recombinant proteins were purified under native conditions and OmpL54 was purified under denaturating conditions with Ni-NTA Agarose (Qiagen) according to the manufacturer's instructions (QIAexpressionist manual).

**Table 1:** Primers for amplification of new *ompL* genes.

*Oligonucleotide*	*Sequence (5′ to 3′)* a	*Gene*
MP13166F	CTGTT**CATATG**CAGCAAAACAATCAGGG	*ompL36*
MP13166R	AGAGA**AAGCTT**AGGTCTAACCGAAATCA	*ompL36*
MP12263F	TGCTT**CATATG**GTTTCGCCGGATCAGA	*ompL37*
MP12263R	GAATA**CTCGAG**ATTTTGTGTTTTTGTAGG	*ompL37*
MP13050F	GCTT**CATATG**CAGGAAGATCTGGATGAA	*ompL47*
MP13050R	GTTAAA**CTCGAG**TTTTTTTGTAGGTTGAG	*ompL47*
MP13491F	TGTT**CATATG**AAAGGGATCCAGTCGATA	*ompL54*
MP13491R	AAAGA**CTCGAG**AGGAGCATTATTGAATTC	*ompL54*

a Restriction sites are indicated in bold type.

### Cellular fractionation of *Leptospira*

Fiocruz L1–130 cultures were fractionated using 1% Triton X-114 as described previously [37], except that 0.5% protease inhibitor cocktail (Cat. #P8849, Sigma-Aldrich) was included in the lysis buffer and 20 mM CaCl_2_ was added to the detergent-soluble fraction prior to phase partitioning. For membrane affinity experiments, total membranes were isolated as described previously [24]. Briefly, 5×10^9^ leptospiral cells were washed twice with 10 mM phosphate buffered saline, pH 7.4 (PBS), containing 5 mM MgCl_2_ and resuspended in 0.9 ml of lysis buffer (10 mM TrisHCl, pH 8.0, 5 mM EDTA, 0.5% protease inhibitor cocktail, Sigma-Aldrich) containing 1 mg/ml of lysozyme. The suspension was incubated for 5 min at 4°C and subjected to three cycles of freezing (−80°C) and thawing (room temperature) with vigorous vortexing. Then DNase I (Sigma-Aldrich) was added to a final concentration of 5 µg/ml and the cell suspension was incubated on ice for 20 min. Membranes were recovered by centrifugation at 16,000×g for 15 min at 4°C and resuspended in 0.5 ml of lysis buffer (without lysozyme). A 100 µl aliquot of the membrane suspension was mixed with 100 µl of either 0.2 M Na_2_CO_3_, 3.2 M urea, 1.2 M NaCl, or lysis buffer and incubated for 15 min at 4°C. The samples were pelleted at 16,000×g for 15 min at 4°C and the supernatants were precipitated with acetone. Each membrane pellet and its supernatant precipitate were resuspended in 50 µl of Novex NuPage sample buffer (Invitrogen, Carlsbad, CA).

### Gel electrophoresis, antibodies and immunoblotting

Protein samples were boiled for 5 min in Novex NuPage sample buffer (Invitrogen) in the presence of 2.5% β-mercapthoethanol and separated through Bis-Tris 4–12% polyacrylamide gradient NuPage gels using the Novex XCell Sure Lock electrophoresis cell (Invitrogen).

The polyclonal rabbit sera specific for the following proteins are described elsewhere: P31_LipL45_ [44], LipL31, ImpL63 [36], OmpL1 [22], LipL41 [45], GroEL [46], LipL46 [47], LipL32 [48], and FlaA1 [49]. For production of polyclonal rabbit serum recognizing OmpL36, OmpL37, OmpL47 and OmpL54, the respective purified recombinant proteins were separated by preparative gel electrophoresis and excised from the gel. New Zealand White rabbits were immunized (Animal Pharm Services, Healdsburg, CA) with 0.2 mg of gel-purified recombinant protein five times over a six-week period, and serum was collected one week after the final injection.

For immunoblotting or biotin ligand blotting, proteins were transferred to a polyvinylidene difluoride (PVDF) Immobilon-P membrane (Millipore, Billerica, MA) and probed with rabbit polyclonal antisera or peroxidase-conjugated streptavidin (GE Lifesciences, Buckinghamshire, England), respectively. Bound antibodies were detected using peroxidase-conjugated anti-rabbit antibodies (GE Lifesciences) and enhanced chemiluminescence reagents according to the manufacturer's instructions (Thermo Scientific, Rockford, IL).

### Cell surface proteolysis of intact *Leptospira* cells

*Leptospira* cultures were harvested by low-speed centrifugation at 2,000×*g* for 7 min at room temperature and gently resuspended in PBS-5 mM MgCl_2_ to a final concentration of 2×10^9^ cells/ml. Proteinase K (Sigma-Aldrich) in proteolysis buffer (10 mM Tris-HCl, pH 8.0, 5 mM CaCl_2_) was added to a final concentration of 12.5 to 100 µg/ml. As a negative control, proteolysis buffer alone was added to the cell suspension. After incubation for 1 h at 37°C, the reactions were stopped by addition of 5 µl of the peptidase inhibitor, phenylmethylsulfonyl fluoride (Sigma-Aldrich) (50 mM in isopropanol). The suspensions were then centrifuged at 9,000×g for 5 min and washed twice with PBS-5 mM MgCl_2_.

### Surface biotinylation

*L. interrogans* Fiocruz L1–130 was grown to the density of 5×10^8^ cells/ml and harvested by low-speed centrifugation at 2,000×*g* for 7 min at room temperature. Cells (2×10^9^) were gently resuspended in 600 µl PBS containing 0.4 mg/ml sulfosuccinimidyl-6-(biotinamido) hexanoate (Sulfo-NHS-LC-Biotin) (Thermo Scientific) and labeled for 1 min after which residual Sulfo-NHS-LC-Biotin was quenched by addition of 50 mM Tris-HCl (pH 8.0) for 5 min at room temperature. Inactivated Sulfo-NHS-LC-Biotin was removed by two washes in PBS. For the preparation of labeled lysates, cells were lysed by three rounds of freeze-thawing and then labeled as described above. To extract labeled proteins, 1×10^9^ bacteria were resuspended in 500 µl of 50 mM Tris-HCl (pH 8.0), 100 mM NaCl, 2 mM EDTA, 0.2% SDS and boiled for 5 min. Biotinylated proteins were then affinity-captured with EZview Red Streptavidin Affinity gel (Sigma-Aldrich) according to manufacturer's instructions.

### Surface immuno-fluorescence assay

*L. interrogans* cultures at densities of 2×10^8^ to 5×10^8^ cells/ml were harvested by low-speed centrifugation at 2,000×*g* for 7 min at room temperature and gently resuspended in PBS-5 mM MgCl_2_. A 1-ml suspension of 5×10^8^ spirochetes was added to each well of Lab-Tek Two-Well Chamber Slides (Nalge Nunc, Naperville, IL) and incubated at 30°C for 80 min to adhere cells. Unbound cells were carefully removed by aspiration and remaining intact bacteria were fixed to the glass slides by incubation for 40 min at 30°C in 2% paraformaldehyde in PBS-5 mM MgCl_2_. As a control to demonstrate antibody recognition of subsurface proteins, spirochetes were permeabilized by fixation with 1 ml of 100% cold methanol and incubation at −20°C for 20 min. Non-specific binding was blocked by incubation of slides at 30°C for 90 min in blocking buffer (Difco *Leptospira* Enrichment EMJH, BD, Sparks, MD). Immune and pre-immune sera (when utilized) were diluted in blocking buffer as follows: OmpL36 1∶100, OmpL37 1∶75, OmpL47 1∶75, OmpL54 1∶50, FlaA1 1∶600, OmpL1 1∶100, LipL46 1∶200, and LipL32 1∶800 and incubated on slides for 1 h at 30°C, after which the slides were washed three times with PBS. Alexa Fluor 488-labeled goat anti-rabbit IgG (Invitrogen/Molecular Probes, Eugene, OR) diluted 1∶2000 and fluorescent nucleic acid stain, 4′6-diamidino-2-phenyl-indole dihydrochloride (DAPI) (Invitrogen/Molecular Probes) diluted to a final concentration of 0.25 µg/ml in blocking buffer were used to detect antibody binding and the presence of spirochetes, respectively. After incubation at 30°C for 45 min, the slides were washed twice with PBS and once with sterile water, then the chambers were removed and slides air-dried for 10 min. ProLong Gold anti-fade mounting medium (Invitrogen/Molecular Probes) was added, a cover slip applied, and the slides were cured overnight in the dark. Cover slips were sealed with nail polish and staining was visualized by fluorescence microscopy with a Zeiss Axioskop 40 (Carl Zeiss, Inc., Jena, Germany).

## Results

### Identification of candidate integral outer membrane proteins from the *L. interrogans* genome

Application of the bioinformatic criteria described in Material and Methods led to the selection of OmpL36 (LIC13166), OmpL37 (LIC12263), OmpL47 (LIC13050), and OmpL54 (LIC13491) for further study, designated according to their apparent molecular mass determined by gel electrophoresis. All four candidates were predicted to be nonlipoproteins with a Signal peptidase I (SPI) cleavage site and to lack a membrane-spanning α-helix following the signal peptide. The number of predicted membrane-spanning β-strands were as follows: OmpL36 (≥8), OmpL37 (≥6), OmpL47 (≥8), and OmpL54 (≥8).

### Analysis of the cellular localization of OmpL36, OmpL37, OmpL47 and OmpL54 by detergent extraction

**Figure 1:**
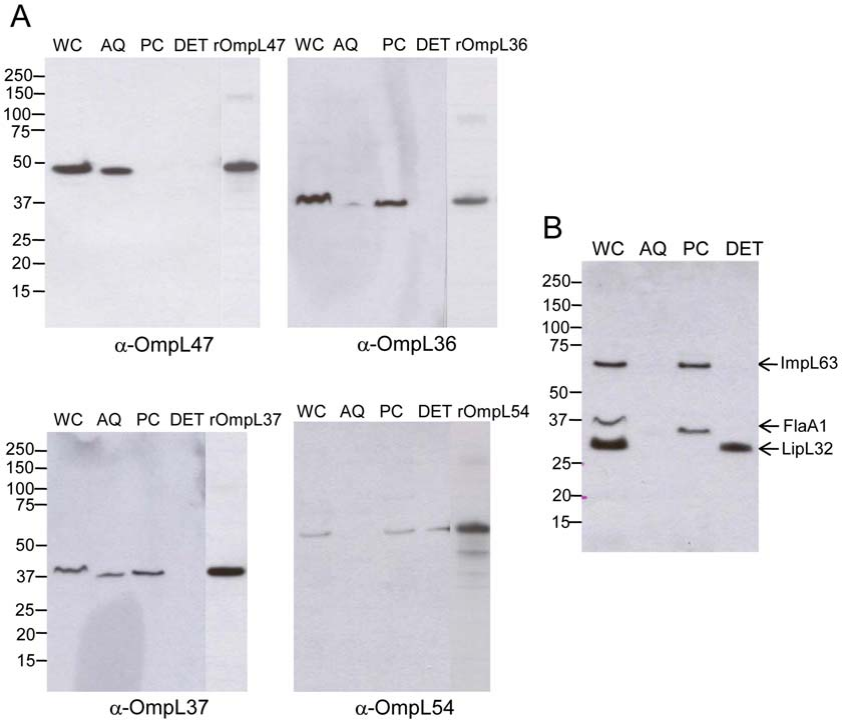
Localization of OmpL36, OmpL37, OmpL47 and OmpL54 after detergent fractionation of *L. interrogans* Fiocruz L1–130. Equivalents of 1×10^8^ of leptospires per lane or 0.5 µg of recombinant proteins per lane are separated on gel electrophoresis (Bis-Tris 4–12% NuPage gel, Novex), blotted to PVDF membrane and probed with rabbit immune sera. *L. interrogans* serovar Copenhageni strain Fiocruz L1–130 whole cell lysate (lane WC), the aqueous fraction (lane AQ), the protoplasmic cylinder fraction (lane PC) and the detergent fraction (lane DET). rOmpL36, rOmpL37, rOmpL47, and rOmpL54 denote the corresponding recombinant proteins. (A) Membranes probed with OmpL36, OmpL37, OmpL47 and OmpL54 antisera. (B) Membrane probed with ImpL63, FlaA1 and LipL32 antisera. The identities of individual proteins are indicated on the right, and the positions of molecular mass standard (in kilodaltons) are indicated on the left.

The expression of OmpL36, OmpL37, OmpL47 and OmpL54 in whole cell lysates of *L. interrogans* serovar Copenhageni strain Fiocruz L1–130 cultivated *in vitro* was confirmed by immunoblot analysis using antisera raised against the respective recombinant proteins (Fig. 1A). Cellular localization was assessed by Triton X-114 detergent solubilization and phase partitioning [50]. This method initially yields two fractions: a detergent insoluble protoplasmic cylinder (PC) fraction and a detergent soluble fraction [37], [39]. The detergent soluble portion is partitioned into two phases by raising the temperature to 37°C, which is above the cloud point of the detergent, resulting in separation of the detergent-rich hydrophobic phase (DET) from the detergent-poor aqueous phase (AQ) [45], [48], [51]. Previous cellular localization studies [37], [39], [45], [48], [51] had reported that leptospiral outer membrane lipoproteins partition to the Triton X-114 detergent-rich phase, while periplasmic proteins separate into the detergent-poor phase and inner membrane and cytoplasmic components are found in the detergent-insoluble fraction.

OmpL36, OmpL37, OmpL47 and OmpL54 were localized by comparing the amounts present in whole cell extracts and Triton X-114 fractions by immunoblot analysis (Fig. 1A and Table 2). Of note, OmpL47 migrates as a 47-kDa band, which is considerably larger than the 39 kDa calculated molecular weight of the protein. Because this is true for both native and recombinant OmpL47, we concluded that the lower electrophoretic mobility is probably due to its low isoelectric point of 5.0, rather than a result of cellular post-translational modification. The mobility of the other three proteins (native and recombinant) corresponded with their calculated molecular weights. Surprisingly, OmpL54 was the only one of the four predicted OMPs detected in the Triton X-114 detergent phase, with most of OmpL54 appearing in the detergent-insoluble fraction (Fig. 1A). OmpL47 was completely solubilized by detergent, but fractionated exclusively into the aqueous phase (Fig. 1A). OmpL36 and OmpL37 were largely detergent insoluble, and the small amount of OmpL37 that was solubilized fractionated into the aqueous phase (Fig. 1A). Analysis of fractions by immunoblot with antisera to reference inner membrane-associated proteins ImpL63 and FlaA1, and the outer membrane lipoprotein, LipL32, confirmed that the Triton X-114 solubilization and fractionation method had been performed correctly (Fig. 1B). The detergent solubilities of the new proteins were further confirmed by extraction with another detergent, 1% Triton X-100. OmpL36 and OmpL37 were Triton X-100 insoluble, OmpL54 was partially solubilized and OmpL47 was completely solubilized by Triton X-100 (data not shown).

**Table 2:** Localization of outer membrane proteins.

	Biotinylation^a^	Proteinase K^b^	Surface IFA^c^	Tx-114^d^	Membrane affinity^e^
**New proteins:**					
OmpL36	++	−	++	−	++
OmpL37	++	++	++	−	+
OmpL47	++	++	++	−*	++
OmpL54	+	++	++	+	++
**Controls:**					
OmpL1	++	Nd	++	+	++
LipL46	++	+	+	++	++
LipL32	++	+	+	++	++
LipL41	++	Nd	Nd	++	++
FlaA1	−	−	−	−	Nd

Applied methods are described in Materials and Methods.

a ++, Protein is extensively biotinylated in intact cells; +, protein is present in slightly higher amounts after biotinylation of lysed cells; −, protein is present in very low amounts or absent after biotinylation of intact versus lysed cells.

b ++, Protein is substantially cleaved by proteinase K (PK); +, protein is cleaved by PK; −, protein remains intact.

c ++, Protein is clearly present on the surface of leptospires; +, protein is present on the surface, but the detection signal is much stronger after membrane has been permeabilized, suggesting only partial surface exposure; −, protein is not detected on the surface.

d ++, Protein is partitioning in detergent phase after Triton X-114 treatment; +, A portion of protein is present in detergent phase −, protein is not partitioning in detergent phase; −*, protein is in aqueous phase.

e ++, Majority of protein is retained in lipid bilayer after treatment with all three different reagents (Na_2_CO_3_, urea, NaCl); +, majority of protein remains with lipid bilayer after treatment with at least two reagents. Nd, not determined.

### Analysis of OmpL36, OmpL37, OmpL47 and OmpL54 by surface proteolysis

**Figure 2:**
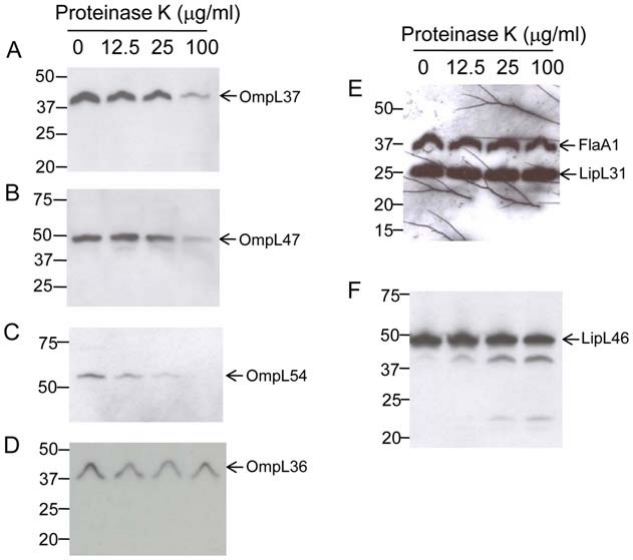
Surface localization of *L. interrogans* serovar Copenhageni strain Fiocruz L1–130 proteins by proteinase K treatment. Whole intact spirochetes were incubated with different concentrations of proteinase K, equivalents of 1×10^8^ of leptospires per lane separated by gel electrophoresis (Bis-Tris 4–12% NuPage gel, Novex), transferred to a PVDF membrane, and probed with polyclonal rabbit antisera against: (A) OmpL37; (B) OmpL47; (C) OmpL54; (D) OmpL36; (E) FlaA1 and LipL31; (F) LipL46. The identities of individual proteins are indicated on the right, and the positions of molecular mass standard (in kilodaltons) are indicated on the left.

The new OmpL proteins were localized by proteinase K treatment of intact leptospires. A range of proteinase K concentrations was tested to determine the conditions for exclusive cleavage of surface proteins (Fig. 2). OmpL37, OmpL47 and OmpL54 were susceptible to protease treatment in a dose dependent manner (Fig. 2A, B, and C), while no detectable cleavage of OmpL36 was observed (Fig. 2D). The subsurface proteins, endoflagellar sheath protein, FlaA1, and the subsurface protein, LipL31, were used as negative controls for surface proteolysis (Fig. 2E). Neither FlaA1 nor LipL31 were digested by any concentration of proteinase K tested on intact leptospires (Fig. 2E). However, when spirochetes were solubilized with Triton X-100 prior to protease treatment, both FlaA1 and LipL31 were completely digested with 100 µg/ml of proteinase K (data not shown). Previously characterized surface lipoprotein, LipL46 [47], was used as a positive control (Fig. 2F). Slight cleavage of LipL46 occurred with smaller cleavage fragments being produced, indicating that only a portion of this lipoprotein is surface-exposed and/or accessible to proteinase K (Fig. 2F). Of note, the relative amounts of LipL46 cleavage products increased with higher proteinase K concentration (Fig. 2F).

### Analysis of OmpL36, OmpL37, OmpL47 and OmpL54 by surface immunofluorescence assay (IFA)

**Figure 3:**
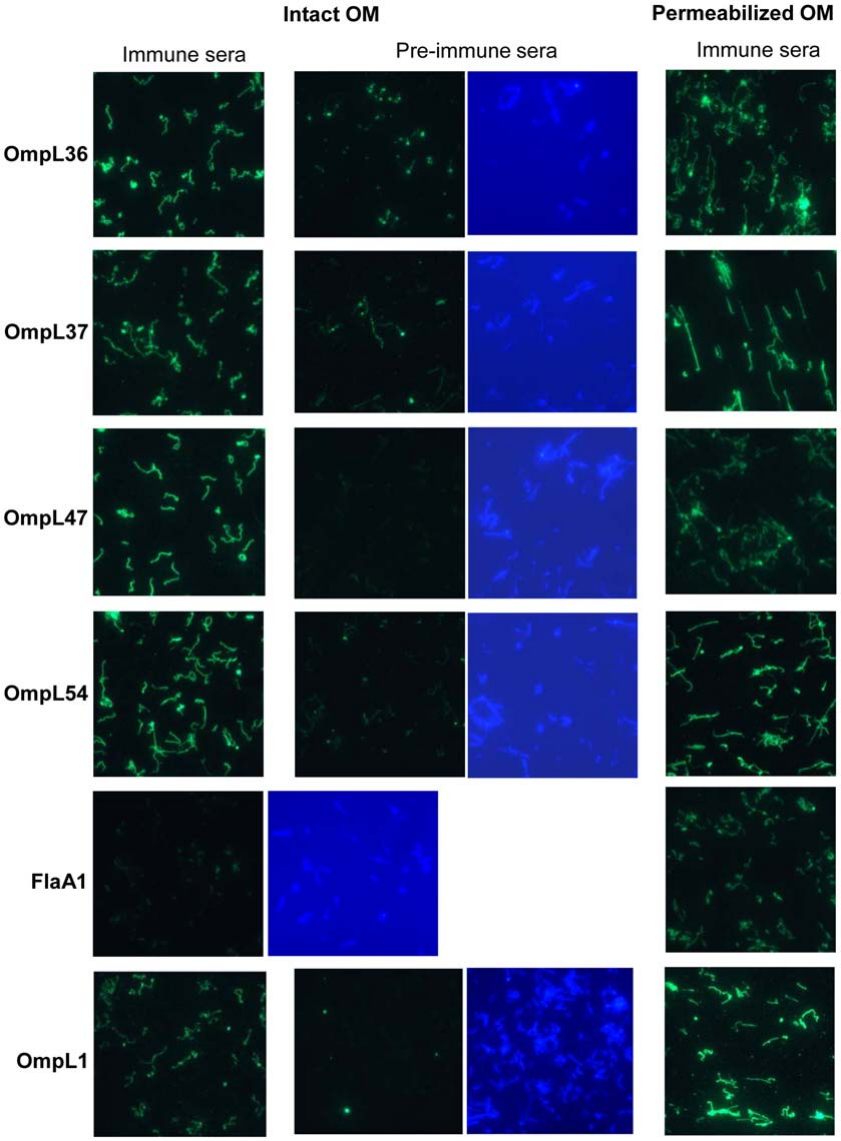
Surface localization OmpL36, OmpL37, OmpL47, OmpL54, and OmpL1 by surface immunofluorescence assay (IFA). Intact or membrane-permeabilized spirochetes were probed with immune and pre-immune sera (when utilized). Binding of rabbit sera to leptospires were detected with Alexa Fluor 488 conjugated goat anti-rabbit IgG fragments. A DAPI counterstain was used to monitor the presence of spirochetes. The identities of individual proteins recognized by the particular antiserum are indicated on the left. All images are taken after 4 sec long exposure.

A surface immunofluorescence assay was used to study the accessibility of proteins to antibody binding to intact vs. permeabilized spirochetes. Leptospires were fixed to glass slides by a low concentration of paraformaldehyde, which leaves the outer membrane intact [27], [52]. Specific immune sera efficiently labeled the surface of intact leptospiral cells (Fig. 3 and Table 2), indicating surface-exposure of OmpL36, OmpL37, OmpL47 and OmpL54. To confirm that the labeling was not the result of a damaged outer membrane, immune serum against the periplasmic flagella component, FlaA1, was used as a negative control. FlaA1 immune serum labeled leptospires only when the cell membranes were permeabilized with methanol prior to antibody addition (Fig. 3). As additional controls, pre-immune sera were tested, excluding the possibility that the observed labeling was due to nonspecific reactivity of rabbit sera with leptospiral surface antigens (Fig. 3). We also investigated the outer membrane-spanning protein, OmpL1, by surface IFA (Fig. 3). OmpL1 in intact leptospires was labeled by immune serum indicating the presence of surface-exposed domains. Somewhat stronger labeling of OmpL1 was obtained when the cells were permeabilized (Fig. 3).

### Surface biotinylation

**Figure 4:**
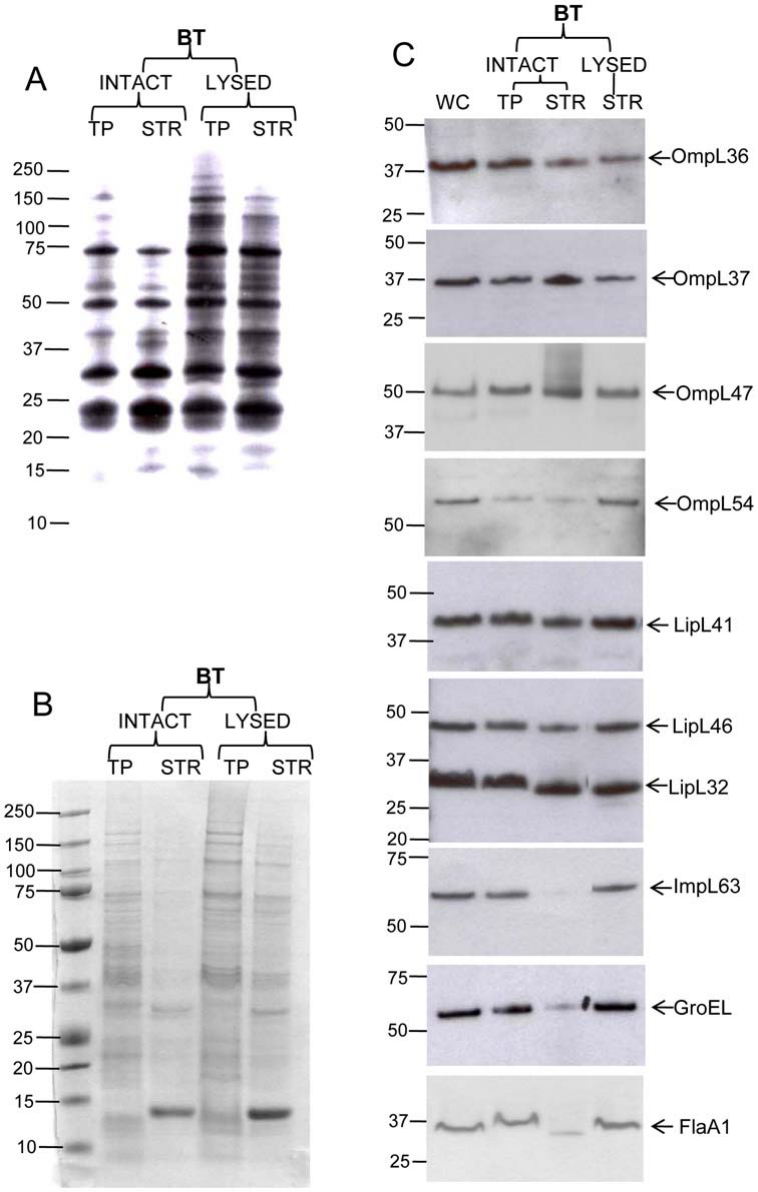
Analysis of biotinylated proteins from intact and lysed *Leptospira.* Proteins of *L. interrogans* serovar Copenhageni strain Fiocruz L1–130 were treated with Sulfo-NHS-LC-Biotin (BT) and equivalents of 1×10^8^ of leptospires per lane were separated on gel electrophoresis (Bis-Tris 4–12% NuPage gel, Novex). A whole cell lysate without Sulfo-NHS-LC-Biotin (lane WC), a total protein of intact (INTACT) or lysed (LYSED) leptospires after biotinylation (lanes TP) and material captured from biotinylated leptospires by streptavidin affinity gel (lanes STR). (A) Streptavidin blot. Proteins were blotted to PVDF membrane and the biotin labeled proteins detected by streptavidin horseradish peroxide (HRP) conjugate. (B) A Coomassie G-250 stained gel of samples described above. (C) Immunoblots with specific rabbit sera. The identities of individual proteins are indicated on the right, and the positions of molecular mass standard (in kilodaltons) are indicated on the left.

Viable, intact spirochetes were labeled with the water-soluble, membrane-impermeable reagent, Sulfo-NHS-LC-Biotin. Biotinylated products were captured by streptavidin, separated by gel electrophoresis and visualized by either biotin ligand blotting (Fig. 4A), Coomassie brilliant G-250 staining (Fig. 4B), or immunoblotting (Fig. 4C and Table 2). Biotin ligand blotting revealed selective biotinylation of a clearly defined subset of antigens in intact cells compared to leptospiral cells disrupted by freeze-thawing (Fig. 4A). The loading of equal amounts of whole-cell proteins in Fig. 4A was confirmed by staining with Coomassie brilliant G-250 (Fig. 4B). The most intensely biotinylated bands from intact leptospires had molecular weights of 21, 32, 50, and 70 kDa, accompanied by less prominent bands with molecular weights of 41, 45, and 55 kDa (Fig. 4A). This pattern of biotinylated proteins was reproducibly observed in several experiments (data not shown). The banding pattern of surface biotinylated proteins we observed is similar to what has been previously described, indicating that the 21 kDa, 32 kDa, 41 kDa, 45 kDa, and 50 kDa bands (Fig. 4A) are most likely LipL21 [53], LipL32, LipL41, LipL46 and Q8F8Q0 (OmpL47), respectively [49]. Since we knew that OmpL47 is biotinylated in intact leptospires [49] and because previous surface biotinylation had revealed several uncharacterized protein bands [53], we investigated whether OmpL36, OmpL37 and OmpL54 are also susceptible to surface biotinylation. Surface-biotinylated samples and samples biotinylated after cell lysis were subjected to immunoblotting with specific antisera (Fig. 4C). OmpL36, OmpL37 and OmpL47 were captured by streptavidin in amounts comparable to the positive control proteins, LipL41, LipL46, and LipL32 (Fig. 4C). Biotinylation of OmpL54 was detected at a low level, and the weakness of the signal could be due to the low expression levels of OmpL54 in *Leptospira* (Fig. 4C). ImpL63, GroEL, and FlaA1 were included as negative controls and showed relatively little capture by streptavidin in the samples from intact cells compared to those from lysed cells (Fig. 4C).

### Membrane affinity analysis

**Figure 5:**
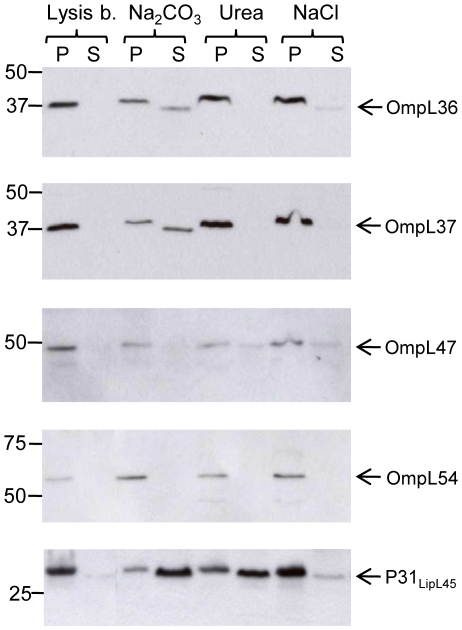
Membrane affinity analysis of OmpL36, OmpL37, OmpL47 and OmpL54. Membrane fraction of *L. interrogans* was treated with lysis buffer as a control or 0.1 M Na_2_CO_3_ (pH 11), 1.6 M urea, or 0.6 M NaCl for 15 min at 4°C. Samples were pelleted by centrifugation to separate the pellets (P) and supernatants (S), followed by gel electrophoresis (Bis-Tris 4–12% NuPage gel, Novex), and immunoblotting with specific antisera. The identities of individual proteins are indicated on the right, and the positions of molecular mass standard (in kilodaltons) are indicated on the left.

To investigate the relationship of the new OmpL proteins with the membrane lipid bilayer, membrane affinity analysis was performed. Treatment of bacterial cells with lysozyme, alternating freezing and thawing, followed by centrifugation separates proteins into soluble (cytoplasmic and periplasmic) and pellet (total membrane) fractions [54]. The membrane fraction was treated under various conditions, including high pH (0.1 M Na_2_CO_3_), high salt (0.6 M NaCl), or urea (1.6 M), to release peripheral membrane proteins not anchored in the lipid bilayer [24], [44], [55], [56]. Immunoblot analysis of the soluble (supernatants) and insoluble (pelleted) membrane fractions revealed that the bulk of the investigated proteins remained associated with the membrane fraction after a high-salt wash (Fig. 5 and Table 2). OmpL36, OmpL37 and OmpL54 were resistant to urea treatment, while a minor portion of OmpL47 was released from the membrane fraction by urea (Fig. 5). OmpL54 was resistant to high pH treatment, whereas small amounts of OmpL36, OmpL37 and OmpL47 were released from the membrane by Na_2_CO_3_ treatment (Fig. 5). The peripheral membrane protein, P31_LipL45_, also known as Qlp42 [57], was included as a positive control, showing substantial release from the membrane by urea and Na_2_CO_3_ (Fig. 5), as previously described [44]. As expected, the integral outer membrane protein OmpL1 could not be released from the membrane by any treatment (Table 2; [24]).

## Discussion

Outer membrane proteins of Gram-negative bacteria are of great interest because of their location on the cell surface where bacterial pathogens interact with the host. In particular, OMPs may play key roles in pathogenesis by acting as (i) adhesins, (ii) targets for bactericidal antibodies, (iii) receptors for various host molecules, and/or (iv) porins. In the case of pathogenic *Leptospira* species, OMPs would be key mediators of the adaptation and response to changes in environmental conditions inside and outside of the host during their life cycle. Leptospiral surface components are thought to mediate interactions with host molecules, counteract host defense mechanisms, and promote the invasion and colonization of various tissues. Therefore, the elucidation of surface-exposed proteins is critical to the understanding of pathogenesis mechanisms, the development of diagnostic antigens, and the identification of potential vaccine candidates. While several surface-exposed lipoproteins and putative lipoproteins, including LipL32 [49], LipL46 [47], LipL41 [45], LipL21 [53], LigB [46], [58], [59], Loa22 [60], [61], Omp52 [62], Lsa21 [63], and Lsa24 [64] have been described in *Leptospira* spp., only one OM-spanning protein, OmpL1, has been well characterized [22], [24], prompting the search for additional OM-spanning proteins in these organisms.

Spirochetes are diderm bacteria with both inner (cytoplasmic) and outer membranes. In conformity with the secondary structure of proteins from Gram-negative bacteria, spirochetal inner membrane proteins are predicted to span the lipid bilayer in α-helical hydrophobic stretches approximately 20 amino acids in length. The identification and membrane topology of inner membrane protein sequences is relatively straightforward to predict using Kyte-Doolittle hydropathy plots and other bioinformatic tools [65]. In contrast, OMPs are thought to lack long hydrophobic stretches because they would cause the protein to be retained in the inner membrane, thus preventing it from reaching the outer membrane [12]. Instead, the crystal structure of transmembrane OMPs (mostly porins) reveal multiple membrane-spanning domains consisting of β-strands arranged in a barrel [66]. The membrane-spanning β-strands are amphipathic, such that the outer face of the β-strand is hydrophobic and interacts with the lipid bilayer and the inner face is hydrophilic and interacts with the aqueous pore of the protein. The topological model of the OmpL1 porin contains ten such amphipathic transmembrane β-strands [22], [24]. A number of genes have been identified in the leptospiral genome that may also encode proteins with amphipathic transmembrane β-strands [15]. Given that *B. burgdorferi* and *T. pallidum* lack LPS on their surface [67], [68], we acknowledge that not all spirochetal integral OMPs may conform to this structural pattern. In fact, a recently described OMP of *T. pallidum*, Tp0453, has been suggested to insert in the OM by amphipathic α-helices and induce membrane permeability [69]. Until recently, α-helices were the only transmembrane secondary structures that could be accurately predicted from novel amino acid sequences with any reasonable degree of confidence [70], [71]. In our study, we exploited two contemporary transmembrane β-sheet prediction programs [18], [19] in conjunction with additional prediction tools used in OMP selection [15], [41] to find potential transmembrane OMPs encoded by the *L. interrogans* serovar Copenhageni strain Fiocruz L1–130 genome. The OmpL36, OmpL37, OmpL47 and OmpL54 proteins met our transmembrane OMP prediction criteria and were further characterized for surface exposure and membrane affinity using multiple complementary experimental methods.

Cellular fractionation by Triton X-114 extraction and phase partitioning has been broadly applied to determine whether or not proteins are in the leptospiral outer membrane [22], [36], [37], [39], [44], [47], [48], [53]. However, this method has had limited validation in the case of OM-spanning proteins, such as channel-forming OMPs (porins), which contain substantial amounts of amphipathic regions that could account for uncharacteristic interactions with Triton X-114 [72]. In fact, a number of clear examples of incomplete detergent solubilization of known leptospiral outer membrane proteins, including the porin, OmpL1, have been described [22], [45], [47], [53], indicating that complete fractionation into the Triton X-114 detergent phase may not occur for transmembrane OMPs and that additional methods are needed to assess the localization of leptospiral proteins. Our Triton X-114 fractionation experiments revealed that only OmpL54 is present to any significant extent in the detergent phase, with OmpL36 and OmpL37 being present mostly in the detergent insoluble (protoplasmic cylinder) fraction, and OmpL47 fractionating exclusively into the aqueous phase (Fig. 1A and Table 2). The unexpected presence of OMPs in the protoplasmic cylinder fraction has been described previously for several leptospiral OMPs: OmpL1 [22], LipL41 [45], LipL21 [53] and LipL46 [47]. The partitioning of OmpL47 selectively to the aqueous phase was unanticipated. However, such partitioning has been described for the eukaryotic channel-forming protein AcChoR [72] and borrelial porins Oms28 and Oms66 (P66) [25], [73]. Based upon prior studies, largely with OM-lipoproteins, the poor solubility of OmpL36, OmpL37 and OmpL54 in Triton detergents would have been interpreted as evidence that these proteins are not OMPs. For this reason, we performed additional localization experiments to investigate whether the unique amphipathic nature of transmembrane OMPs (as opposed to OM-lipoproteins) could account for their differential solubility in Triton detergents.

*In situ* proteolysis studies on intact cells were conducted to determine whether our OmpL proteins are localized on the surface of *Leptospira* (Fig. 2 and Table 2). Proteinase K is a relatively non-specific protease cleaving accessible parts of proteins, such as those exposed on the surface of intact cells, and for this reason is a broadly used method to examine the surface exposure of proteins in *Borrelia* [26], [29], [74]–[76], *Treponema* [69], [77]–[80] and other bacteria [81]–[84]. Previous efforts at proteolytic cleavage of surface-exposed leptospiral proteins either have not been successful [49] or not appropriately designed due to lack of controls and a high concentration of proteinase K used [62]. For example, Loa22 is a surface-exposed OMP [61] that is insensitive to proteinase K cleavage [60], and cleavage of Omp52 is inconclusive due to the excessive concentration of the enzyme used and the lack of surface or subsurface controls [62]. We adapted this method to leptospiral cells by testing a range of proteinase K concentrations from 12.5 to 100 µg/ml, using known surface (LipL46) and subsurface (FlaA1 and LipL31) proteins as positive and negative controls, respectively. OmpL37, OmpL47 and OmpL54 were found to be susceptible to proteinase K cleavage, indicating their surface exposure. Cleavage of the OmpL36 protein could not be detected, and cleavage of the positive control, LipL46, was incomplete, suggesting that proteinase K cleavage sites may have been inaccessible perhaps due to steric hindrance by LPS at the cell surface [49]. These data indicate that proteinase K may not be able to digest all leptospiral surface proteins and requires confirmation by complementary surface exposure assessment methods.

The surface immunofluorescence assay is a well-established and highly sensitive method to investigate the surface exposure of bacterial proteins [26], [49], [52], [61]. Surface IFA was utilized to determine whether the new OmpL proteins are exposed on the surface of intact *Leptospira*. The surface IFA technique described here is similar to that used to demonstrate the surface exposure of borrelial [26], [52] and other leptospiral [49], [61] proteins. The surface IFA method was adapted to minimize the manipulation of cells in an effort to maintain outer membrane integrity while taking advantage of the ability of *Leptospira* to adhere to glass slides. We used a lower concentration (2% versus 4%) of paraformaldehyde and fewer washing steps than previous studies [49], [61]. Surface IFA showed the labeling of leptospiral cells using antisera against OmpL36, OmpL37, OmpL47 and OmpL54 (Fig. 3 and Table 2). FlaA1 was efficiently labeled only when the outer membrane was permeabilized, confirming the integrity of the leptospiral outer membrane. Antiserum to OmpL1 was included in surface IFA studies as a positive control. The slightly stronger labeling of OmpL1 in spirochetes with permeabilized outer membranes might be due to antibodies not efficiently recognizing the native epitopes of OmpL1 and/or the fact that the majority of the protein is integrated into the lipid bilayer. The surface exposure of our OmpL proteins shown by surface IFA prompted us to perform additional, confirmatory studies.

Surface biotinylation has been widely used to identify bacterial surface antigens [49], [52], [53], [84]. Biotin labeling of intact *Leptospira* results in the selective biotinylation of a distinct subpopulation of proteins referred to as the leptospiral “surfaceome”, including LipL21 [53], LipL32, LipL41 and Q8F8Q0 [49]. Affinity capture of biotinylated proteins from intact cells revealed that OmpL36, OmpL37 and OmpL47 are present on the surface of *Leptospira*, while the levels of OmpL54 biotinylation are too low to interpret with confidence (Fig. 4C and Table 2). The surface biotinylation results with OmpL47 were consistent with the previous “surfaceome” study in which OmpL47 was referred to as Q8F8Q0 [49].

Next, we investigated whether the new OmpL proteins are integral or peripheral membrane proteins. We applied several membrane affinity methods whereby leptospiral membranes are fractionated by treatment with reagents designed to release peripheral membrane proteins not integrated into the lipid bilayer. Membrane affinity methods have been previously utilized to assess the membrane integration of OmpL1, LipL41 and P31_LipL45_ [24], [44]. P31_LipL45_ was determined to be a peripheral membrane protein because urea and high pH released the protein from leptospiral membranes [44]. It should be noted that this method does not differentiate between inner membrane and outer membrane proteins. The new OmpL proteins were not significantly released from membranes by a high salt concentration, indicating that electrostatic charge is not the primary mode of membrane association. OmpL36, OmpL37 and OmpL54 were completely resistant to urea treatment, with a small fraction of OmpL47 being released by urea. Minor fractions of our OmpL proteins were released by high pH, but not to the extent of the peripheral membrane protein, P31_LipL45_, which was included as a positive control (Fig. 5). The transmembrane protein, OmpL1 [22], [24], was included as negative control and was found to remain membrane-anchored despite treatment of the membranes with urea, high salt, or high pH (Table 2). It should be noted that small amounts of known OM-lipoproteins, LipL41, LipL46 and LipL32, were also released from the membrane by high pH ([44] and data not shown). It should also be noted that, although most of the integral outer membrane proteins of *E. coli* are alkali insoluble [54], [56], OmpA is an exception [54], supporting our view that the behavior of outer membrane proteins in various methods is complex and that localization studies should include a variety of experimental methods.

A multi-faceted approach using independent methods is essential for determining a transmembrane OMP's location based on the following criteria: 1. Predicted structure; 2. Surface exposure; and 3. Membrane integration. Bioinformatic analysis of potential transmembrane OMPs should demonstrate an amino-terminal export signal peptide (lacking a lipoprotein signal peptide lipobox) and at least 6 membrane-spanning β-strands without multiple alpha-helical transmembrane domains. Experimental requirements should be satisfied using multiple methods both for membrane integration and surface exposure as summarized in Table 2 for OmpL36, OmpL37, OmpL47 and OmpL54. It should be noted that our results for three of these proteins are further supported by the finding that homologues of OmpL36 (AAN51159), OmpL37 (AAN48694), and OmpL47 (AAN47704) are present in outer membrane vesicles of a clinical isolate of *L. interrogans* serovar Copenhageni [38].

In conclusion, we employed five independent experimental methods to examine transmembrane OMPs: Triton X-114 fractionation, surface proteolysis, surface immunofluorescence, surface biotinylation, and membrane affinity analysis. These methods were used to characterize four novel leptospiral proteins that are both surface-exposed and membrane-integrated, leading to the conclusion that these proteins are transmembrane OMPs, which we have designated OmpL36, OmpL37, OmpL47, and OmpL54. Our findings further indicate that the Triton X-114 method of cellular fractionation may not be appropriate for the localization of transmembrane OMPs. The failure of the Triton X-114 method to correctly fractionate leptospiral transmembrane OMPs may also have important implications for studies on transmembrane OMPs of other spirochetes. We also describe the proteinase K treatment as improved and applicable method to assess the surface exposure of leptospiral transmembrane OMPs. We believe that our studies provide a path from genomic sequence data to the elucidation and characterization of OMPs, particularly integral outer membrane-spanning proteins. Our approach of employing *in silico* analysis to select the potential integral OMPs and subsequent experimental validation with a panel of experimental techniques is a direct and effective strategy for the identification of novel surface-exposed antigens that have the potential to serve as diagnostic antigens and vaccine candidates. Future studies are planned for structural and functional characterization of these transmembrane OMPs to determine their roles in the biology of and pathogenicity of *Leptospira* species.
